# Supplementary material for: Evaluation of the Spray Drying Conditions of Blueberry Juice-Maltodextrin on the Yield, Content, and Retention of Quercetin 3-d-Galactoside
Source: Polymers (Basel). 2019 Feb 13;11(2):312. doi: 10.3390/polym11020312 (PMC6419557; doi:10.3390/polym11020312)
Supplement: Supplementary file 1 [file polymers-11-00312-s001.pdf]

polymers

Supplementary Materials

Evaluation of the Spray Drying Conditions of Blueberry Juice-Maltodextrin on the Yield, Content and Retention of Quercetin 3-D-galactoside

María Z. Saavedra–Leos[1], César Leyva-Porras[2]*, Laura A. López-Martínez[3], Raúl González-García[4], Joel O. Martínez[4], Isaac Compeán Martínez[1] and Alberto Toxqui-Terán[5]

1 Coordinación Académica Región Altiplano, Universidad Autónoma de San Luis Potosí. Carretera Cedral Km. 5+600 Ejido San José de las Trojes Matehuala, S.L.P. C.P. 78700. México, zenaida.saavedra@uaslp.mx (M.Z.S.-L.), isaac.compean@uaslp.mx (I.C.M.)
2 Centro de Investigación de Materiales Avanzados S.C. (CIMAV), Miguel de Cervantes No. 120, Complejo Industrial Chihuahua. Chihuahua, Chih. C.P. 31136. México, cesar.leyva@cimav.edu.mx (C.L.-P.)
3 Coordinación Académica Región Altiplano Oeste, Universidad Autónoma de San Luis Carretera Salinas – Santo Domingo 200, C.P. 78600 Salinas de Hidalgo, S.L.P. México, araceli.lopez@uaslp.mx
4 Facultad de Ciencias Químicas, Universidad Autónoma de San Luis Potosí, Av. Dr. Manuel Nava 6, San Luis Potosí, S.L.P., C.P. 78210, México, raulgg@uaslp.mx (R.G.-G.); atlanta126@gmail.com (J.O.M.)
5 Centro de Investigación de Materiales Avanzados (CIMAV-Mty), Alianza Norte No 202, Parque de Investigación e Innovación Tecnológica, C.P. 66600, Apodaca Nuevo León, México, alberto.toxqui@cimav.edu.mx (A.T.-T.)
* Correspondence: cesar.leyva@cimav.edu.mx; Tel.: +52 (614) 4391-106.

S1. Thermal analysis of selected BJ-MX samples.

Figure S1 shows the TGA and DSC curves for some selected Blueberry juice-Maltodextrin (BJ-MX) samples. From the TGA, a humidity content of 2%–5% at 125 °C, and the onset of thermal degradation at about 160 °C, was observed. The TGA showed the presence of several degradation events in the range of 160–425 °C, indicated by the changes in the slope of the curve. At 425 °C and above, the BJ-MX was fully carbonized.

The DSC showed three endothermic events, the first related to the evaporation of water in the temperature range of 50–125 °C, the second identified as the melting of low molecular weight sugars at 130–175 °C, and the third corresponding to the melting of high molecular weight sugars such as the maltodextrins at 175–250 °C. Each of the two melting events was followed by the thermal degradation of the low and high molecular weight sugars, and content in the BJ-MX, respectively. However, the XRD results clearly showed the absence of diffraction peaks, indicating that powders remained in the amorphous state.

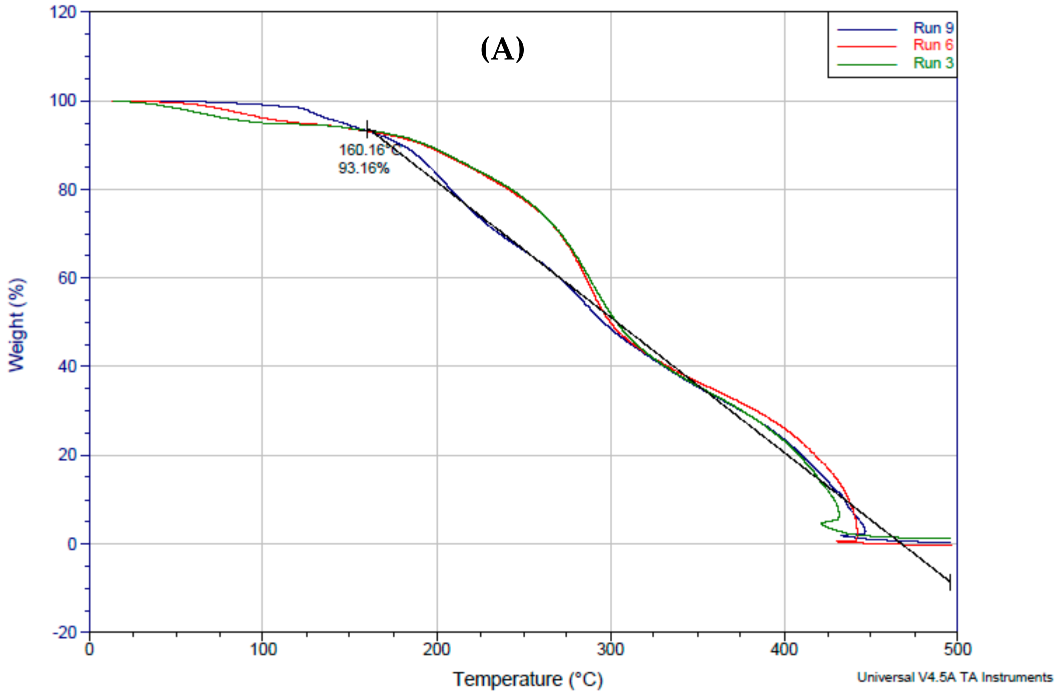

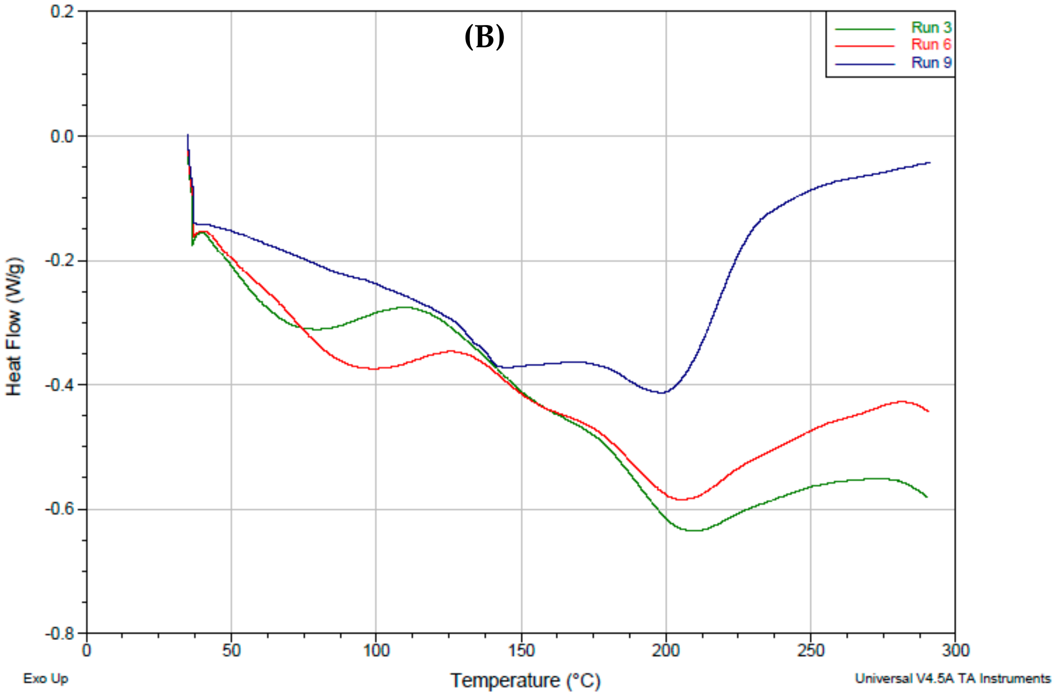

Figure S1. Thermal analysis of selected Blueberry juice-Maltodextrin (BJ-MX) samples. A) TGA and B) DSC.

S2. Antioxidant quantification by HPLC

For the quantification of the antioxidant content in the dry samples, 0.5 g of powder was dissolved in 0.5 ml of phosphoric acid (10% v/v in water), and 3 ml of methanol as extracting solvent. Solution was stirred by 5 minutes, and left to rest by 24 hours in darkness in order to maximize the extraction of the antioxidants. After the time had elapsed, the solution was filtered in an Acrodisc filter (0.45 μm). The filtered solution was diluted with 200 μL of methanol, and then a constant volume of 10 μL was injected in the HPLC instrument. Injections were carried out by triplicate.

The antioxidant content of quercetin 3-D-galactoside was quantified by high performance liquid chromatography (HPLC) with a Waters system (Waters Corp. Milford MA, USA), equipped with a binary pump, an auto-injector (model 717), and a dual wavelength absorbance detector (model 2487). The analyses were carried out at room temperature, and pH of 3.0. A constant flow rate of 1 ml/min solution of 50% acetonitrile–phosphoric acid was employed as the mobile phase. Detection was set at 306 nm. The chromatographic separation was done with an Agilent C-18 column (75 x 4.6 mm DI 3.5 μm). All data were analyzed with the Empower Pro software (Version 4.0) (Waters Corp. Milford MA, USA).

The calibration curves were constructed employing a quercetin 3-D-galactoside HPLC grade standard (>97.0%, Sigma-Aldrich). A stock solution of 1000 μg/ml was prepared, and from this solution, several aliquots (0.01, 1, 5, 10, and 20 μg/ml) were prepared in order to obtain the calibration curve. The calibration curves were prepared the same day of the injection in the HPLC. Figure S2 shows the chromatograms obtained for a calibration curve of quercetin 3-D-galactoside. The elution time of the antioxidant was 3.1 min, while the mobile phase eluted at 0.92 min. The intensity of the peak at 3.1 min linearly increased with the concentration of the antioxidant. After the integration of the area under the curve of the elution peak at 3.1 min, the data were plotted versus the concentration. Figure S3 shows the corresponding calibration curve and the repetitions. On the plots, the corresponding equation and the R-squared value were included, indicating the repeatability, and the closeness of the experimental data to the regression line, respectively. Table S1 summarizes the experimental data obtained for each calibration curve and the corresponding repetitions. The average values from the linear regression (m, b and R^2) were employed for calculating the concentration of the antioxidant in the dry powders. Figure S4 shows a typical chromatogram of an injected sample from a dry powder (experiment 18). Additionally to the mobile phase and antioxidant peaks, other peaks at elution times of 1.5 and 2.5 minutes were observed, which may correspond to the methanol added during the extraction process, and some other impurities contained in the juice. After integrating the area under the curve, the content of the antioxidant was determined from the comparison with the calibration curve.

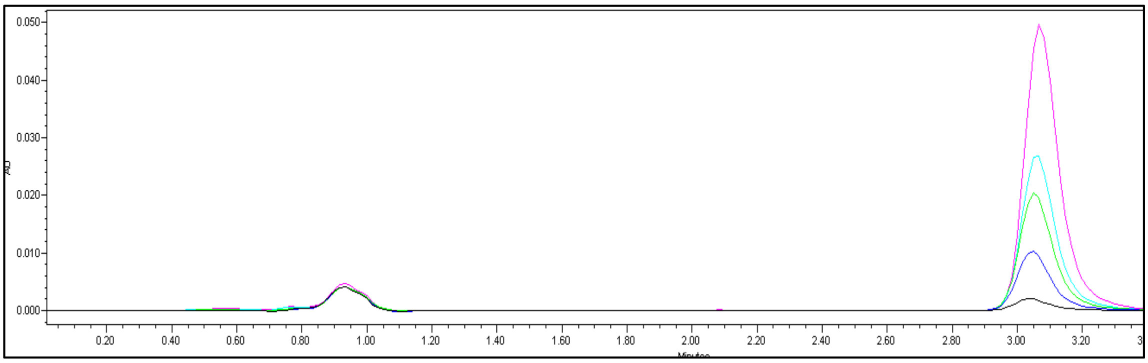

Figure S2. Chromatograms obtained for the calibration curve of quercetin 3-D-galactoside at five concentrations (0.1, 1, 5, 10, and 20 μg/ml).

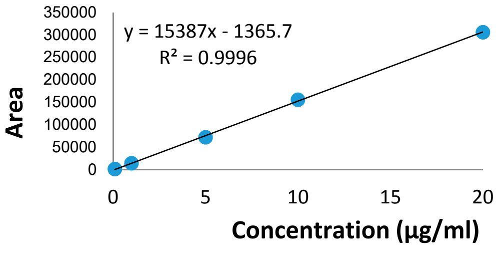

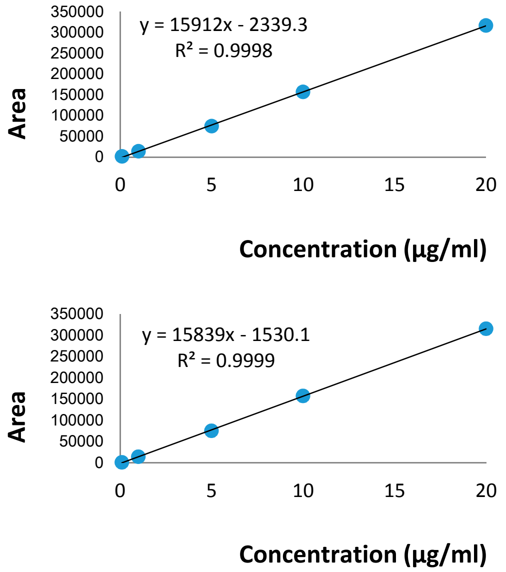

Figure S3. Calibration curves for quercetin 3-D-galactoside.

Table S1. Experimental data obtained for each calibration curve.

CONCENTRATION (µg/ml)	AREA (a.u.)			AVERAGE	STANDARD DEVIATION
	R1	R2	R3		
**0.1**	1371.0	1276.0	1363.0	1336.67	52.69
**1**	13971.0	13837.0	14450.0	14086.00	322.27
**5**	72064.0	74283.0	75426.0	73924.33	1709.46
**10**	155466.0	156665.0	157336.0	156489.00	947.34
**20**	305756.0	316680.0	315546.0	312660.67	6006.44
**Linear regression**					
**m**	15386.6	15912.4	15838.5	15712.50	284.65
**b**	-1365.7	-2339.3	-1530.1	-1745.03	521.17
**R^2**	0.9998	0.9999	0.9999	0.9999	0.0001

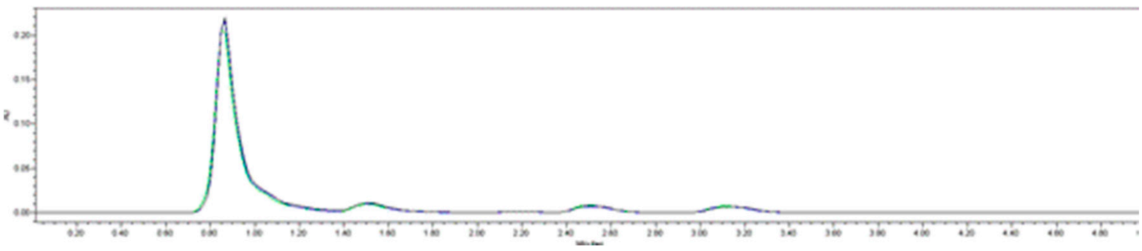

Figure S4. Typical chromatogram of an injected dry powder (experiment 18) after the extraction process.

© 2019 by the authors. Submitted for possible open access publication under the terms and conditions of the Creative Commons Attribution (CC BY) license (http://creativecommons.org/licenses/by/4.0/).
